# Assessment of the Mechanical Properties of ESD Pseudoplastic Resins for Joints in Working Elements of Concrete Structures

**DOI:** 10.3390/ma13112426

**Published:** 2020-05-26

**Authors:** Dominik Logoń, Krzysztof Schabowicz, Krzysztof Wróblewski

**Affiliations:** Faculty of Civil Engineering, Wrocław University of Science and Technology, Wybrzeże Wyspiańskiego 27, 50-370 Wrocław, Poland; krzysztof.schabowicz@pwr.edu.pl (K.S.); k.wroblewski@wp.pl (K.W.)

**Keywords:** ESD resin, expansion joint, quasi-plastic material, energy absorption

## Abstract

Concrete structure joints are filled in mainly in the course of sealing works ensuring protection against the influence of water. This paper presents the methodology of testing the mechanical properties of ESD pseudoplastic resins (E-elastic deformation, S-strengthening control, D-deflection control) recommended for concrete structure joint fillers. The existing standards and papers concerning quasi-brittle cement composites do not provide an adequate point of reference for the tested resins. The lack of a standardised testing method hampers the development of materials universally used in expansion joint fillers in reinforced concrete structures as well as the assessment of their properties and durability. An assessment of the obtained results by reference to the reference sample has been suggested in the article. A test stand and a method of assessing the mechanical properties results (including adhesion to concrete surface) of pseudoplastic resins in the axial tensile test have been presented.

## 1. Introduction

**Figure 1:**
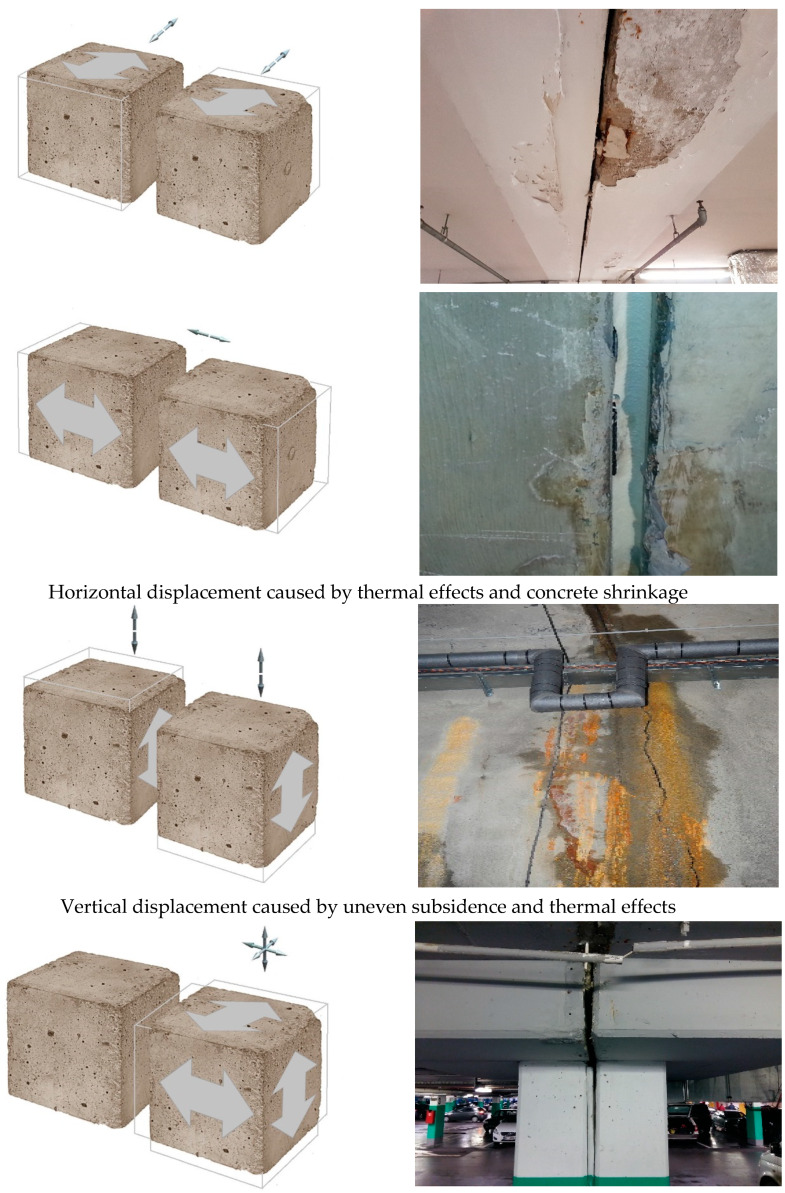
Models showing possible displacements of structural members at expansion joint.

Expansion joints in building structures move in various directions. The most frequent direction of displacement is the direction perpendicular to the edge of the expansion joint. Depending on temperature changes, we observe either the opening, expansion or the narrowing, and closing down of the expansion joint gap [1,2,3]. The widening of the expansion joint gap directly causes the elongation of the material filling in the joint, and if the strength limits of the filler material are exceeded, it results in the irreversible damage causing the loss of sealing reliability. The increasing width of the expansion gap may also result in the filler material breaking off from the concrete surface, which also causes the loss of water tightness. The material can be fed by gravity pouring or by means of pressure injection pumps. The reacting resin hardens and sets, becoming permanently flexible mass, which during the cyclic work of the expansion joint should expand or shrink depending on the changes in expansion joint width, as shown in Figure 1.

Numerous papers have emphasised the dependence of physical and mechanical properties of tested resins on ambient temperature [4,5,6]. The discrepancy of the obtained results has also been attributed to the impact of resins’ adhesion to materials [7]. It has been found that the substrate’s humidity has a significant influence on test results, which makes it necessary to ensure strict control of the testing conditions [8,9]. A review of relevant literature shows that resin tests are carried out on various test stands [10,11,12], which indicates the need to modify the standards to correspond to the scope of conducted tests. The lack of standardised procedures taking into account the possibility of assessing properties makes it impossible to compare the results obtained at various research centres [13,14,15].

The materials showing elastic properties are subjected to tests that provide data regarding the maximum elongation of the material in question or the maximum breaking force. A lot of standards enable the basic test to be carried out, i.e., the axial tensile test [16,17,18,19,20,21,22,23,24,25,26]. It is worth noting that none of the current standards is dedicated to expansion joint quasi-plastic filler materials, and the existing methods of assessment of the adhesion to the substrate do not refer to pseudoplastic behaviour under load.

In the course of research [27,28], an analysis has been conducted with respect to curves obtained in a static tensile test taking into account multi-axial stress and strain of elastic materials. The paper [29] described a methodology of tests of elastomeric bearings in complex states of strain according to the requirements of the standards PN-EN 1337-3 [30] and PN-ISO 37 [16]. The authors presented a design for a modern stand for biaxial tensile test for elastomers exceeding the scope of application of the aforementioned standards. The conducted tests cannot be adapted for the purposes of assessment of mechanical properties of pseudoplastic resins intended to be used as expansion joint fillers, including their adhesion to the concrete substrate.

The method of conducting tests and assessment of the deformation capacity dedicated to quasi-brittle cement composites that is proposed in numerous standards and papers [31,32,33,34,35,36] is not sufficient for the assessment of the mechanical properties of pseudoplastic resins functioning as expansion joint fillers. The existing hyperelastic models for the description of the behaviour of non-elastic materials (including hyperelastic resins) do not, however, take into account the impact of a large number of variables determining the usefulness of ESD materials (E-elastic deformation, S-strengthening control, D-deflection control) as expansion joint fillers. There is no information on the adhesion of materials to concrete substrates, and in particular on the strengthening control and deflection control areas [37,38].

The method proposed in [31,32,33] for the assessment of ESD quasi-brittle cement composites has been used in our own work. Those works present a possibility of assessing the mechanical properties of materials based on the stress–strain correlation in any case of the loads recording the values (force, deformation and absorbed energy), but do not include information regarding changes in the width of gaps in expansion joints during an axial tensile test.

In this work, the aforementioned method has been modified for the purpose of assessing the mechanical properties of ESD quasi-plastic resins in the tensile strength tests—taking into account the assessment of their adhesion to the concrete surface.

## 2. Testing

### 2.1. Materials Used for Tests

In this article, the following specimens has been prepared for testing the mechanical properties of ESD pseudoplastic resins recommended as concrete structure joint fillers:

- specimen 0C—standard, reference cement mortar with the following composition and parameters: cement CEM I 32.5, compressive strength *f_c_* = 4.48 MPa, mortar class M4, three-point bending tensile strength *f_tb_* = 0.14 MPa (distance between supports 150 mm).
- resins used as a filler in the expansion joint model, as presented in Table 1:
  1A—resin based on thixotropic acrylic for injections,
  2A—resin based on acrylic mixed with water,
  3A—resin based on polyurethane mixed with water,
  4A—polyurethane-based resin,
  5A—resin based on elastic epoxy,
  6A—bitumen-based resin.

As shown in Table 1, six types of resins have been selected for tests. Those resins are universally used and available on the construction market and, more importantly, are used as expansion joint fillers. Each resin is characterised by different properties and technical parameters. The resins were fed into the gaps of the expansion joint model by means of the so-called gravity pouring. Subsequently, the expansion joint models were put aside until the resins hardened, i.e., for a period of 24 h. The entire testing process was divided into several series of tests. Each series of tests was conducted for a different resin. In total, tests for six different filler resins were carried out.

The axial tensile test for resins has been conducted in accordance with PN-EN ISO 37 standard [16] using the Instron 33R strength testing machine.

**Figure 2:**
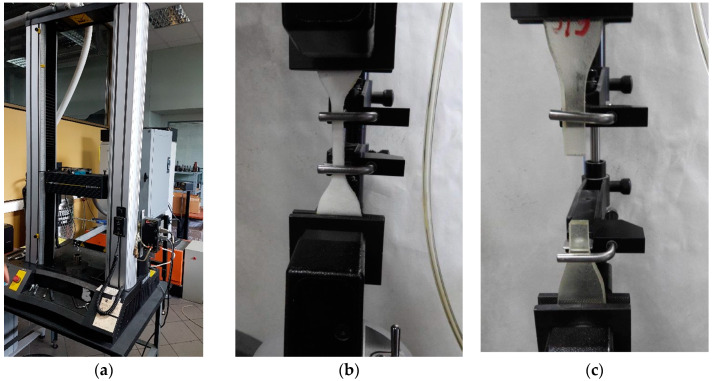
Resin testing according to [16]—axial tensile test: (**a**) view of the strength testing machine—test stand, (**b**) view of the shaped resin elements, the so-called “oars”, placed in the clamps and (**c**) view of the completed test at the moment of breaking of the resin specimen.

The testing procedure included the proper preparation of specimens. Resins in Table 2 were placed on a flat surface 400 × 300 mm, circa 5 mm thick. Afterwards, with the use of a template, shaped elements were cut out—the so-called “oars”—which were then placed, one by one, in the strength testing machine, as shown in Figure 2.

The testing was carried out until the moment of breaking of the resin specimens for which the results of maximum elongation at break and maximum breaking force were obtained.

**Figure 3:**
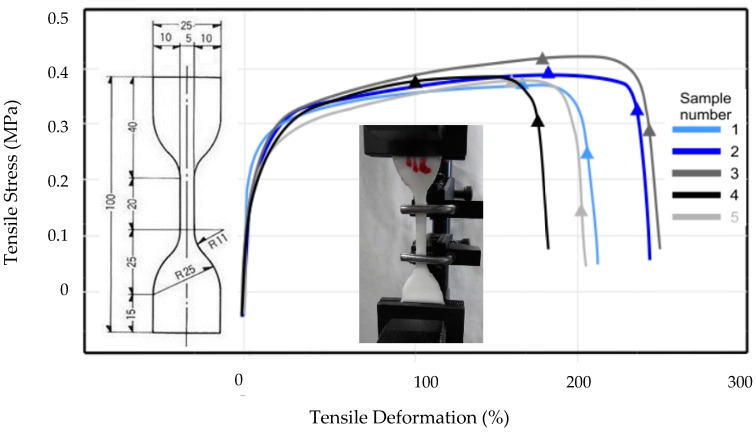
The tensile stress-deformation curves as a function of deformation in axial tensile test of resin 1, Table 2 (5 samples - based on thixotropic acrylic) according to PN-EN ISO 37 [16].

Examples of diagrams of stress as a function of deformation in axial tensile test—5 samples of resin 1 (Table 2) are presented in Figure 3.

The obtained results of the axial tensile test for the analysed resins according to PN-EN ISO 37 [16] are presented in Table 2.

### 2.2. Preparation of Specimens for Tests

**Figure 4:**
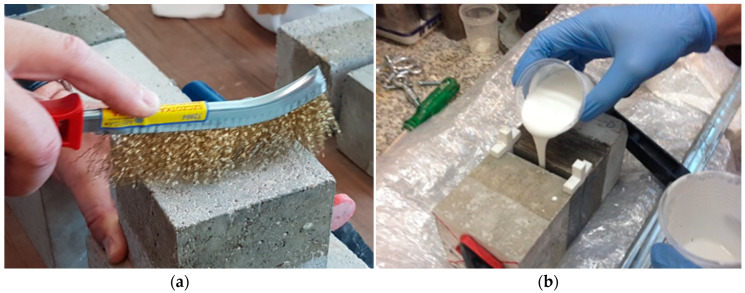
Preparation of a specimen for tests: (**a**) cleaning the surface and (**b**) pouring the resin into the expansion joint.

The expansion joint model prepared for the tests was a model in which class C37 concrete specimens with 100 × 100 × 100 mm dimensions were used. The tests were carried out in laboratory conditions, with a temperature of 20 °C and stable air humidity. The preparation of the expansion joint model consisted in arranging two concrete specimens in parallel with each other, Figure 4.

For all expansion joint model specimens, a 10-mm-wide gap was prepared, and each of the resins and the reference cement mortar was then poured into that gap. In the case of all the fillers, there was the same method of preparation - mechanical cleaning of the concrete specimens surface (1A,2A,3A,4A,5A,6A–Table 1) and pouring the resin into the expansion joint. 

### 2.3. Description of the Test Stand

**Figure 5:**
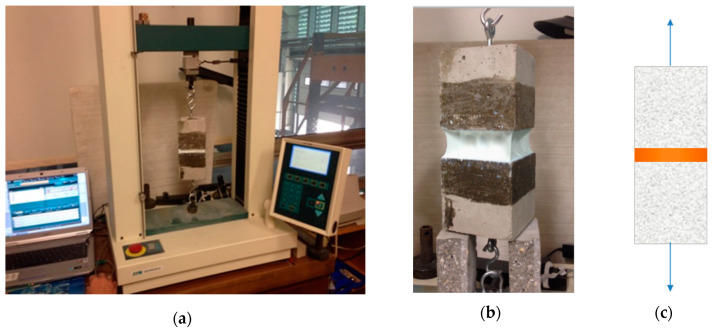
A view of the test stand: (**a**) testing machine, (**b**) close-up of a specimen during testing and (**c**) the adopted tension method.

The expansion joint model specimens were tested using a Hounsfield 10K-S strength testing machine (Tinius Olsen TMC-United States) and the Horizon numerical processing software (ver.1). The adopted crosshead speed was 5 mm/min. A view of the test stand is presented in Figure 5.

Owing to the use of clamps on a harness (jointed system)—the expansion joint model displacement in one direction, perpendicular to the side surfaces of the gap, was simulated. The expansion joint model tension reflects the actual behaviour of filler materials in repaired reinforced concrete structures. The displacements in perpendicular direction are smaller and are not the main cause of destruction of pseudoplastic fillers in expansion joints.

The aim of the tests of expansion joint models filled with sealing resins was to determine the force–deformation correlation serving as the basis for the assessment of the usefulness of resins as expansion joint fillers. The test was based on the measurement of the displacement of crosshead. The stress–strain tests took into account the weight of the lower concrete cube. After the test, the resin condition after the break and the percentage degree of the resin adhesion to the concrete substrate were assessed visually.

**Figure 6:**
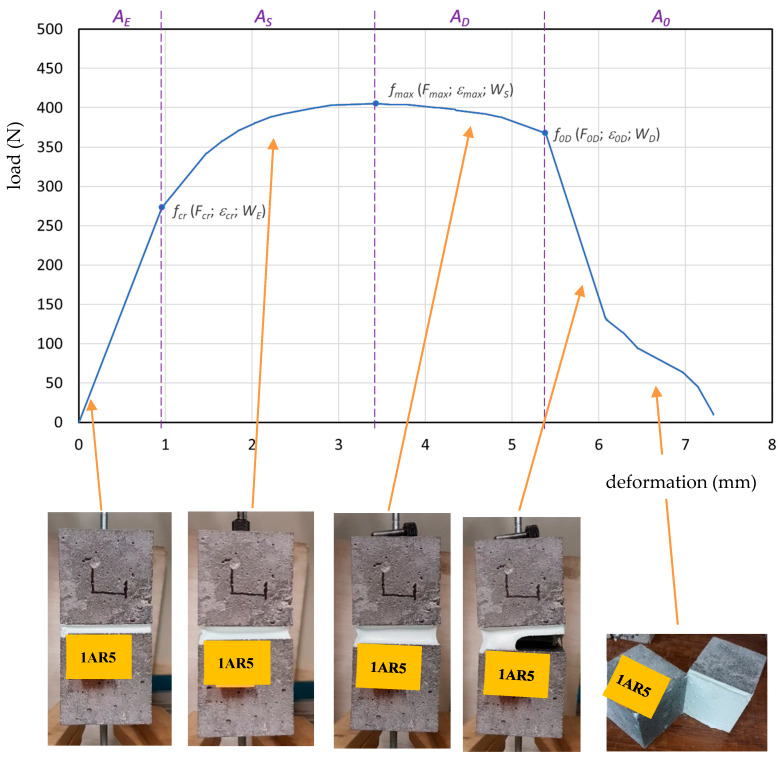
An exemplary diagram of the load–deformation curve for an expansion joint model specimen AR5 with photographs depicting the testing process—manual cleaning surface.

Figure 6 shows an exemplary load–deformation curve for an expansion joint model specimen marked as 1AR5 (resin 1, humidity or contamination of the substrate A: dry, substrate cleaned manually R, specimen 5) with photographs depicting the testing process. Manual cleaning concrete surface results in less resin adhesion (than mechanical cleaning).

### 2.4. Diagram of Properties Assessment for ESD Pseudoplastic Materials

**Figure 7:**
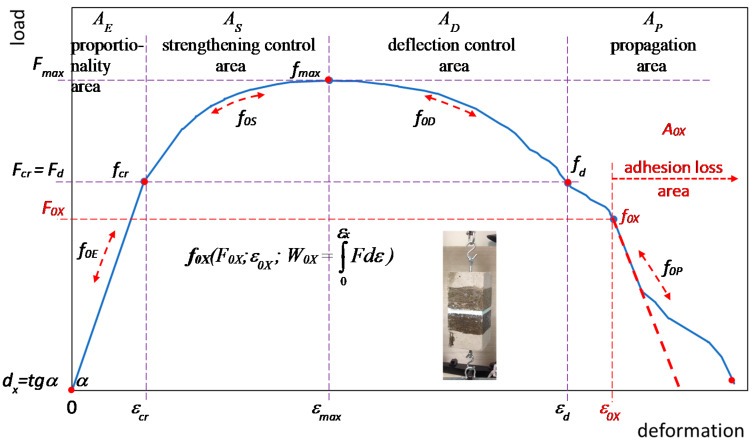
A diagram for the assessment of mechanical properties based on the load–deformation curve of ESD pseudoplastic materials in an axial tensile test.

Figure 7 shows a diagram for the assessment of mechanical properties based on the load–deformation curve of ESD pseudoplastic materials (*E*-elastic deformation, *S*-strengthening control, *D*-deflection control) in an axial tensile test according to [31,32,33].

The drawing outlines the adhesion loss area *A_0X_* in which the resin has been pull off from the concrete surface.

Characteristic areas have been determined: *A_E_*-elastic deformation (*f_cr_*, the proportionality range-Hooke’s law), *A_S_*-strengthening control (the area between *f_cr_*, and the occurrence of maximum stress *f_max_*), *A_D_*-deflection control (the area between *f_max_* and *f_d_)* and *A_P_*-propagation area.

Point *f_d_* corresponds to the ability to carry stress *f_cr_* and is a determinant of the optimum ESD pseudoplastic filler.

Any point *f_X_*(*F_X_,ε_X_,W_X_*) on the load–deformation curve has been defined with the use of a corresponding force *F_X_*, deformation *ε_X_* and absorbed energy *W_X_* (area under the curve).

The characteristic points *f_X_* ending each of the areas *A_X_* marked respectively: *f_cr_, f_max_, f_d_.*

What has been identified is the pulling off or breaking off of the pseudoplastic filler at any point *f_0X_*(*F_0X_,ε_0X_,W_0X_*) and the correlating area *A_0X_* (point *f_0E_* in the proportionality area *A_0E_*, point *f_0S_* in the strengthening area *A_0S_* and point *f_0D_* in the deflection control area *A_0D_*—additionally point *f_0P_* in the propagation area *A_0P_*).

Point *f_0X_*, where the pull off/destruction of the material was recorded, was considered to be the end point of the deformation capacity assessment. If there was no pulling off the resin, point *f_d_* ending the deflection control range served as the determinant of the end of the test. 

The assessment of the deformation capacity of the materials used in the Hooke’s law range has been determined as *d_x_* = *tgα*.

## 3. Test Results

**Figure 8:**
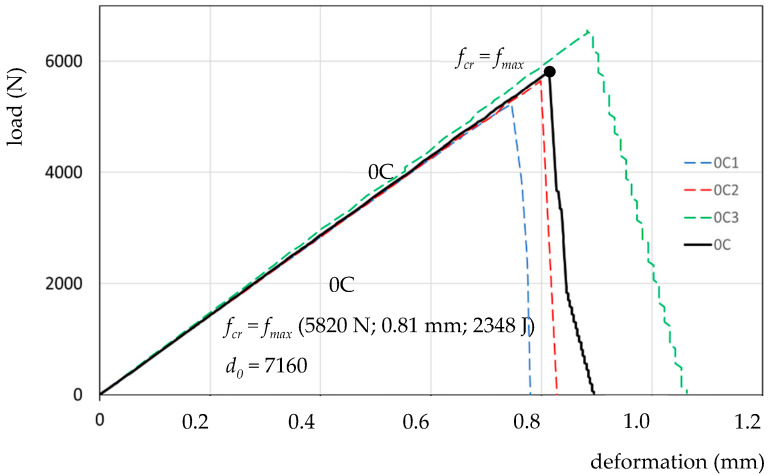
Presentation of load–deformation curves for expansion joint model specimens 0C1, 0C2, 0C3 and the reference specimen 0C in the axial tensile test.

The presentation of the results of 0C1, 0C2, 0C3—a model with M4 cement mortar filler-and the averaged result serving as the reference 0C for the tested resins, is shown in Figure 8.

For the averaged reference specimen *0C*, the following have been determined: *f_cr_*(force-*F_max_*; deformation-*ε_max_*; absorbed energy-*W_max_*), and deformation capacity *d_0_*. Also *f_cr_ = f_max_* (5820 N; 0.81 mm; 2348 J) and *d_0_* = 7160 (tgα force-deformation correlation from the Hooke’s law range) have been determined. The results are presented in Table 3.

**Figure 9:**
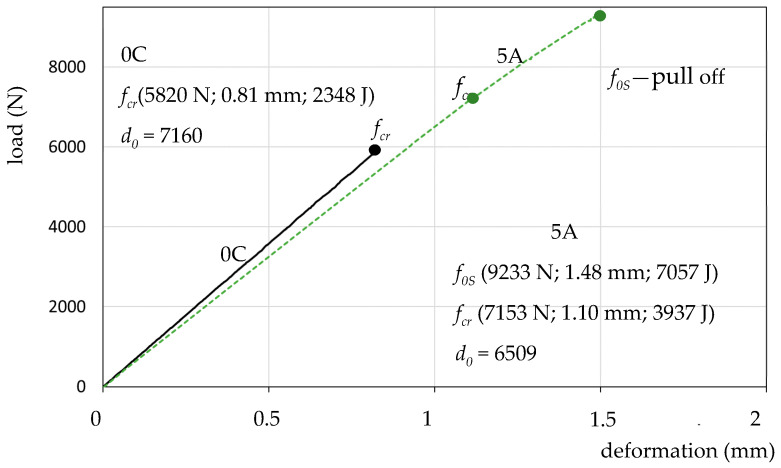
The load–deformation curve for specimen *5A* and specimen *0C*.

Figure 9 shows a diagram for the reference specimen and the “strongest” resin 5A (based on elastic epoxide). Specimen 5A shows slightly higher deformation capacity *d_0_* = 6509 (lower tgα than the reference specimen *d_0_* = 7160), a larger proportionality area *f_cr_* and, additionally, a significant strengthening control area to point *f_0S_*, at which there was a catastrophic, rapid break of the resin.

**Figure 10:**
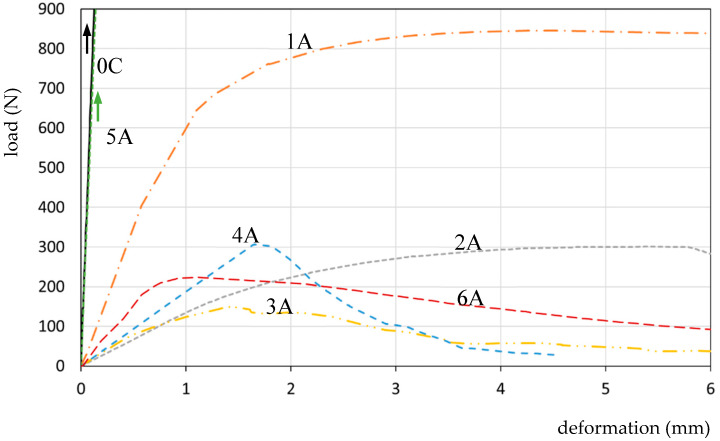
The load–deformation curves for specimens 1A, 2A, 3A 4A 5A, 6A and the reference specimen 0C.

Figure 10 presents collective curves for resins 1A, 2A, 3A, 4A, 5A, 6A and the reference specimen *0C*. Each resin was tested on at least three specimens, out of which the most representative one was selected for comparison purposes. It was not necessary to average the diagrams due to the deformation of the moment of resin destruction.

For each of the presented resins, characteristic points *f_X_* and *f_0X_* (moment of destruction/pulling off the resin) were determined, which is shown in Table 3.

The obtained data show more precisely the behaviour of expansion joint filler materials in a tensile test, enabling the assessment and comparison of various resins. The presented data enable the assessment of the behaviour of resins in each of the proportionality, strengthening control and deflection control areas. A tabular presentation of data enables the characteristic points *f_x_* from a number of tests to be averaged for the purpose of the assessment of ESD pseudoplastic materials.

**Figure 11:**
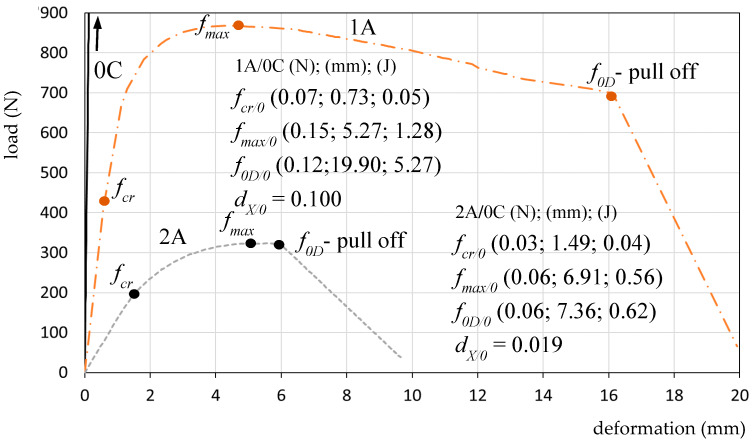
The load–deformation curves for specimens 1A, 2A and specimen 0C.

Figure 11 is a presentation of load–deformation curves for the reference specimen 0C and resins 1A, 2A, comparing the obtained values of mechanical properties with the defined reference specimen.

Table 4 contains a comparison of the obtained values of the mechanical properties of the tested resins 1A, 2A, 3A, 4A, 5A and 6A in relation to the reference specimen 0C.

Table 5 presents the results of resins 1A, 2A, 3A, 4A, 5A, and 6A compared to the linear correlation of specimen *5A*, with the largest Hooke’s law area.

The proposed method of describing points on the force–deformation curve enables the comparison of any selected points *f_x1_* (or areas *A_X1_*) and their comparison with any selected points *f_x__2_* (areas *A_X__2_*) chosen for the analysis of the obtained effects: *f_x__1_/f_x__2_(F_X__1_/F_X__2_,ε_X_ 1/ε_X__2_,W_X__1_/W_X__2_)*.

The results presented in Table 4 and Table 5 indicate the possibility of juxtaposing freely the data selected for analysis, enabling the comparison of multiples of the achieved effects on various test stands. However, it is recommended to quickly introduce a standardised testing procedure that will enable an accurate comparison of the results obtained at various research centres.

**Figure 12:**
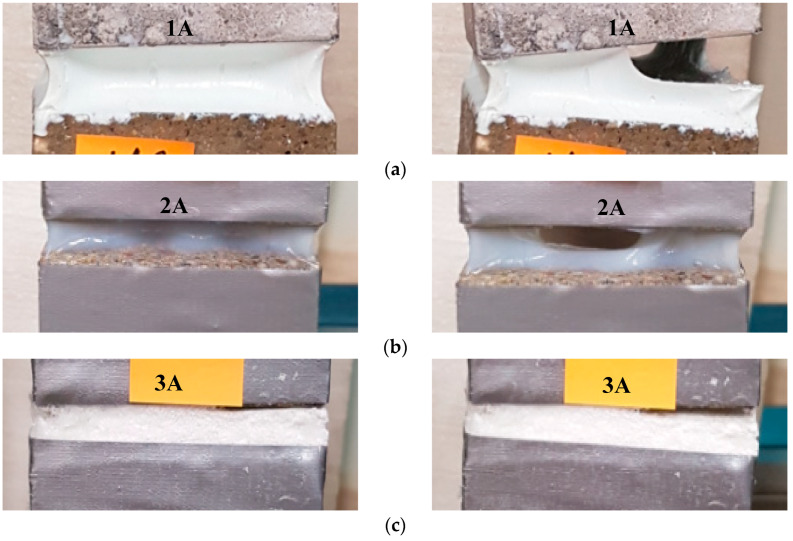
Examples of specimens of resins filling the expansion joints before and after the loss of adhesion to the surface (point *f*_0*X*_) in an axial tensile test: (**a**) resin 1A, (**b**) resin 2A and (**c**) resin 3A.

Examples of deformations of resins 1A, 2A and 3A filling the expansion joints before and after the loss of adhesion to the substrate (point *f_0X_*) in an axial tensile test is presented in Figure 12.

A linear decrease in the load–deformation correlation in an axial tensile test of the presented model indicates the pulling off the fillers from the substrate. That process may be more or less dynamic. In the case of resin 5A, there was a catastrophic break.

Resins 2A and 1A were characterised by a less rapid loosening process—as seen in the diagram. Resins 4A, 3A and 6A, after a partial loosening, were characterised by the greatest capacity for deformation and energy absorption in the destruction propagation area. In that area, the loss of tightness of the joints occurs, which is why that range is not taken into consideration when interpreting the obtained results.

## 4. Discussion of the Results

The assessment of tensile tests of a model of an expansion joint filled with ESD resin mass is made possible by the recording of data: force/stress, corresponding deformation and work-as the quantity of absorbed energy (surface area under the stress–strain curve), Figure 7. The characteristics of each point *f_x_* on the load–deformation curve are presented in the form of *f_x_* (force/load; deformation; absorbed energy), Figure 8, Figure 9, Figure 10 and Figure 11.

The proportionality area *A_E_* determines the quality of the filler, whereas the areas of strengthening control *A_S_* and deflection control *A_D_* (characterised by a greater ability to carry stress than the Hooke’s law proportionality area) contribute to the improvement of durability of sealants in the expansion joints of concrete structures, and constitute an additional safety range.

The characteristics of ESD pseudoelastic resins serving as sealants in expansion joint gaps indicate an increased deformation capacity in areas *A_S_* and *A_D_*—resulting in a considerable quantity of absorbed energy compared to the proportionality area *A_E_*. The strengthening control area *A_S_* is more significant in that type of fillers (it does not generate a destruction of the filler structure).

The deflection control area *A_D_*, with loads greater than those occurring in the proportionality area and comparable to those in the strengthening control area, additionally enables an effective implementation of the applicability range of the ESD resin material in expansion joints. That effect is also controlled by the possible appearance of the first forms of destruction of the structure on the side surface of the pulled material (it usually cannot be seen with a naked eye). 

The area of propagation, weakening *A_P_*, with the decreasing stress and increasing deformation, is characterised by the moment of the resin specimen being pulled off from the concrete surface together with the absorption of another portion of energy in the resin structure destruction process. The area of propagation (of carrying stress that is lower than the critical one) is not recommended for ESD pseudoplastic sealing resins (it may also be assessed).

The area of the loss of adhesion *A_0_* (Figure 7), with the decreasing stress and increasing deformation, is characterised by the moment of the resin specimen being pulled off from the concrete surface together with the destruction of the resin structure. The deformations breaking and destroying the structure of the material result in a significant linear decrease in stress (linear decrease in the force–deformation correlation). What determines the maintenance of reliability of the whole system, i.e., the expansion joint gap filled with sealing resin, is the adhesion control area *A_0_*. The loosening of the resin from the concrete surface is considered an emergency situation. The loss of adhesion of the sealing resin in the expansion joint is, from the perspective of the maintenance of the sealant’s reliability, the moment when water tightness is lost. At that moment, the water influencing the whole system is able to permeate through the filled expansion joint. 

From the perspective of the sealant’s reliability, it is assumed that the loss of adhesion should occur as late as possible. The breaking off, loosening of ESD pseudoplastic resins should take place in the propagation area *A_P_*, which is not significant as regards the values of carried stress. 

What is an important component of the analysis of the obtained results is the correct determination of the Hooke’s law range (*f_cr_*). The linear correlation should be determined on large-scale diagrams. It should be noted that the correlation should be determined from a section between 50% *f_cr_* and *f_cr_*. The force-deformation correlation in the initial range is not linear due to the specimen’s arrangement on the slings and, therefore, should not be taken into account (when interpreting the test results).

A need to standardise the testing procedure has been emphasised. Because the tests are conducted on differing test stands (e.g., with different crosshead speed, ambient temperature, etc.), the obtained data cannot be compared with one another. Until relevant standard provisions are adopted, it is proposed to compare the results as a multiple of the established reference item. It may be a specimen with defined parameters or e.g., the Hooke’s law range of the best of the tested materials.

The obtained results of the tests of resins presented in Table 3 enable a preliminary assessment of the possibility of using them as expansion joint fillers. Depending on the obtained deformations, an appropriate filler can be selected for a specific case of an expansion joint on a structural element. Thus:

- If the expansion joint gaps expand by 5% in relation to the original width, then practically all the resins can be used as fillers for such a joint. All resins 1A, 2A, 3A, 4A, 5A and 6A meet that condition in area *A_E_*.
- If the expansion joint gaps expand by 10% in relation to the original width, then practically all the resins can be used as fillers for such a joint. Resins 2A, 4A and 5A meet that condition in area *A_E_*, and resins 1A, 3A and 6A—in area *A_S_*.
- If the expansion joint gaps expand by 15% in relation to the original width, resin 5A cannot be used to fill such a joint. Resins 1A, 2A. 3A and 4A meet that condition in area *A_S_*, and resin 6A—in area *A_D_*.
- If the expansion joint gaps expand by 50% in relation to the original width, resins 3A, 4A, 5A and 6A cannot be used to fill such a joint. Resin 2A meets that condition in area *A_S_*, and resin 1A—in area *A_D_*.
- If the expansion joint gaps expand by 100% in relation to the original width, then only resin 1A can be used as a filler for such a joint. Resin 1A meets that condition in area *A_D_*.

A comparison of the tested resins with the reference specimen 0C (Table 4) confirms the conclusions presented above. An analysis of the results in relation to a predefined reference item, based on a multiple of changes, enables a comparison of the results obtained at various research centres—until an applicable standard (norm) is established. In summary, it should be noted that only specimens 1A and 2A could be classified as ESD pseudoplastic resins. Specimen 1A shows lower deformation capacity (d_x_ = 715) in comparison with specimen 2A (d_x_ = 135), with a much greater ability to carry stress and higher quantity of absorbed energy in the areas: elasticity, strengthening control and deflection control. 

What is worth noting is the comparison of the results of the proposed testing method with the traditional method of assessing the mechanical properties of resins presented in Figure 3 and Table 2. The presented data show that resin 1 (Figure 3) has different ESD mechanical parameters compered to resin 1A (Figure 11), which leads to various conclusions regarding the assessment of the usefulness of pseudoplastic materials proposed as fillers in working elements of concrete structures—taking into account the assessment of their adhesion to the concrete surface.

The comparison of Figure 11 and Figure 6 shows that mechanical cleaning of the concrete surface 1A results in better resin adhesion than manual preparation of the concrete surface 1AR5.

As mentioned above, the existing hyperelastic models used for a description of the correlation of non-elastic materials (including hyperelastic resins) do not take into account the impact of a large number of variables determining the usefulness of ESD materials as expansion joint fillers. In particular, they do not take into consideration the materials’ adhesion to concrete surfaces and the strengthening control and deflection control areas.

## 5. Conclusions

An assessment has been proposed of the mechanical properties of ESD pseudoplastic materials for working joints of concrete structures, by means of a description *f_X_* (force, deformation and absorbed energy) on a force–deformation curve, in an axial tensile test.

It has been suggested that there is a need for a standardised test stand together with a reference specimen to enable a comparison of the obtained values of mechanical properties with a multiple of the established reference specimen. Such an assessment enables a comparison of the results of tests carried out at various research centres.

Quasi-plastic ESD fillers are recommended for joints in working concrete structures. The proportionality area (the range of stress, deformation and absorbed energy) determines the quality of the filler, whereas the strengthening control and deflection control areas contribute to the improvement of durability of concrete fillers and serve as an additional protection for sealants.

For ESD materials, it is not recommended to assess the destruction propagation area as that range generates too large deformation of pseudoplastic materials—with abilities to carry stress lower than *f_cr_* (Hooke’s law range).

Based on the proposed tests and analyses, it has been found that resin 1A—based on thixotropic acrylic—is the best ESD pseudoplastic specimen.

## Figures and Tables

**Table 1:** Resin specimens—tensile strength *f_t_* elongation at break ε, density, viscosity and pH.

Specimen	Tensile Strength *f_t_* (MPa)	Elongation at Break *ε* (%)	Density (kg/dm^3^)	Viscosity (mPas)	pH
1A	>0.4	>240	0.95–2.09	<50	5–11
2A	>0.6	~50	0.99–2.09	<50	5–11
3A	>0.6	>80	0.99–1.16	<200	ca. 7
4A	ca. 0.7	ca. 50	0.99–1.10	80–155	-
5A	>4.0	ca. 70	0.97–1.11	500–1700	-
6A	ca. 0.2	ca. 500	0.99–1.10	1750–3500	-

**Table 2:** Results from the axial tensile test for the analysed resins according to PN-EN ISO 37 [16].

Tested Resin	Specimen No.	Load at Maximum (N)	Tensile Stress at Maximum (MPa)	Load at Break (N)	Tensile Stress at Break (MPa)	Tensile Strain for *F_max_* (%)	Tensile Strain at Break (%)
Resin 1	1	10.56	0.406	8.066	0.310	199.7	241.8
	2	10.90	0.425	9.626	0.376	201.2	257.7
	3	10.86	0.430	7.969	0.316	179.5	246.0
	4	10.41	0.421	8.237	0.33	157.4	236.3
	5	11.08	0.420	5.437	0.206	193.7	236.5
Average values		10.76	0.421	7.867	0.308	186.3	243.7
Resin 2	1	6.77	0.529	2.106	0.165	6.5	37.8
	2	7.88	0.737	2.409	0.225	1.1	17.3
	3	6.13	0.605	2.289	0.203	3.4	23.8
Average values		6.93	0.624	2.268	0.198	3.67	26.3
Resin 3	1	28.88	0.716	23.457	0.582	174.0	175.3
	2	28.62	0.664	8.435	0.196	162.3	160.3
	3	28.22	0.681	23.259	0.561	167.7	186.2
	4	27.22	0.623	24.968	0.571	186.3	197.3
	5	28.28	0.743	25.995	0.683	170.4	178.1
Average values		28.24	0.685	21.223	0.519	172.1	179.4
Resin 5	1	481.29	6.398	481.286	6.398	115.2	115.2
	2	466.59	5.938	466.588	5.938	102.2	102.2
	3	446.46	5.539	215.259	2.670	89.7	89.1
	4	468.19	5.792	467.858	5.788	96.7	96.9
	5	450.42	5.980	450.136	5.976	103.6	103.8
Average values		462.59	5.929	416.226	5.354	101.5	101.4

**Table 3:** Presentation of the results (characteristic points on the force–deformation curve) for the reference specimen 0C and tested resins 1A, 2A, 3A, 4A, 5A, 6A.

Specimen	Point	*F*	*ε*	*W*	*d_x_*
		(N)	(mm)	(J)	(N/mm)
*0C*	*f_cr_*	5820	0.81	2348	7160
*1A*	*f_cr_*	424	0.59	124	715
	*f_max_*	868	4.27	3005	-
	*f_0D_*	699	16.12	12370	-
*2A*	*f_cr_*	163	1.21	98	135
	*f_max_*	324	5.60	1323	-
	*f_0D_*	323	5.96	1456	-
*3A*	*f_cr_*	93	0.57	26	165
	*f_0S_*	173	1.57	164	-
*4A*	*f_0E_*	330	1.77	291	186
*5A*	*f_cr_*	7153	1.10	3937	6509
	*f_0S_*	9233	1.48	7057	-
*6A*	*f_cr_*	203	0.64	63	311
	*f_max_*	247	1.17	189	-
	*f_d_*	203	2.95	603	-

**Table 4:** Resins 1A, 2A, 3A, 4A, 5A and 6A compared with the reference specimen *0C*.

Specimen	*f*_*x*/__0_	*F*_*x*/__0_	*ε*_*x*/__0_	*W*_*x*/__0_	*d*_*x*/__0_
1A/0C	*f* *_cr/_*_0_	0.07	0.73	0.05	0.100
	*f* *_max/_*_0_	0.15	5.27	1.28	-
	*f_0D/_*_0_	0.12	19.90	5.27	-
2A/0C	*f_cr/_*_0_	0.03	1.49	0.04	0.019
	*f_max/_*_0_	0.06	6.91	0.56	-
	*f_0D/_*_0_	0.06	7.36	0.62	-
3A/0C	*f_cr/_*_0_	0.02	0.70	0.01	0.023
	*f_0S/_*_0_	0.03	1.94	0.07	-
4A/0C	*f_0E/_*_0_	0.06	2.19	0.12	0.026
5A/0C	*f_cr/_*_0_	1.23	1.36	1.68	0.909
	*f_0S/_*_0_	1.59	1.83	3.01	-
6A/0C	*f_cr/_*_0_	0.03	0.79	0.03	0.043
	*f_max/_*_0_	0.04	1.44	0.08	-
	*f_d/_*_0_	0.03	3.64	0.26	-

**Table 5:** Resins 1A, 2A, 3A, 4A, 5A and 6A compared to the linear correlation of specimen 5A.

Specimen	*f_x/cr_*	*F_x/cr_*	*ε_x/cr_*	*W_x/cr_*	*d_x/cr_*
1A/5A	*f_cr__1/cr_*	0.059	0.536	0.031	0.110
	*f_max__1/cr_*	0.121	3.882	0.763	-
	*f_0D__1/cr_*	0.098	14.655	3.142	-
2A/5A	*f_cr__1/cr_*	0.023	1.100	0.025	0.021
	*f_max__1/cr_*	0.045	5.091	0.336	-
	*f_0D__1/cr_*	0.045	5.418	0.370	-
3A/5A	*f_cr__1/cr_*	0.013	0.518	0.007	0.025
	*f_0S__1/cr_*	0.024	1.427	0.042	-
4A/5A	*f_0E/cr_*	0.046	1.609	0.074	0.029
5A/5A	*f_cr__1/cr_*	1.000	1.000	1.000	1.000
	*f_0S__1/cr_*	1.291	1.345	1.792	-
6A/5A	*f_cr/cr_*	0.028	0.582	0.016	0.048
	*f_max/cr_*	0.035	1.064	0.048	-
	*f_d/cr_*	0.028	2.682	0.153	-
